# Time trends and COVID-19 post-pandemic changes in physical activity and sedentary behavior prevalence among Brazilian adults between 2006 and 2021

**DOI:** 10.1590/1980-549720230011.supl.1

**Published:** 2023-04-21

**Authors:** Thania Mara Teixeira Rezende Faria, Alanna Gomes da Silva, Rafael Moreira Claro, Deborah Carvalho Malta

**Affiliations:** I Universidade Federal de Minas Gerais, School of Medicine, Post Graduate Program in Public Health – Belo Horizonte (MG), Brazil.; II Universidade Federal de Minas Gerais, School of Nursing, Graduate Program in Nursing – Belo Horizonte (MG), Brazil.; III Universidade Federal de Minas Gerais, School of Nursing, Department of Nutrition – Belo Horizonte (MG), Brazil.; IV Universidade Federal de Minas Gerais, School of Nursing, Department of Maternal-Infant Nursing and Public Health – Belo Horizonte (MG), Brazil.

**Keywords:** Physical activity, Sedentary lifestyle, Noncommunicable diseases, Time series, COVID-19, Atividade física, Comportamento sedentário, Doenças não transmissíveis, Séries históricas, COVID-19

## Abstract

**Objective:**

To analyze time trends and prevalence of physical activity and sedentary behavior among adults of Brazilian capitals between 2006 and 2021, including the pandemic period.

**Methods:**

This is a time-series of cross-sectional surveys based on the National Surveillance System for Risk and Protective Factors for Chronic Diseases by Telephone Survey. Trends of sufficient leisure-time physical activity, sufficient physical activity while commuting, insufficient practice of physical activity, and total screen time were estimated by using Prais-Winsten regression. Annual prevalences and time trends were estimated for each indicator by sex, age group and education.

**Results:**

For total population, significant time trends were found for leisure-time physical activity (β=0.614) and total screen time (β=1.319). As for prevalence, leisure-time physical activity increased from 29% in 2009 to 39% in 2019, followed by a reduction of 2.3% between 2020 and 2021. Total screen time prevalence increased considerably between 2019 and 2020 (4.7%). Though physical inactivity tended to reduce along the series, its prevalence increased by 3.4% between 2019 and 2021, as well as physical activity while commuting decreased by 3,7% in the same time period.

**Conclusion:**

Whereas leisure-time physical activity increased over the years, it is uncertain whether this trend will be the same in the years following COVID-19. Not only did people alter their leisure-time habits, but also there was an increasing dominance of screen time due to the change in work and social patterns. More strategies need to be addressed to tackle physical inactivity and sedentary behavior, and to review the post-pandemic national targets.

## RESUMO

**Objetivo:**

Analisar as tendências temporais e a prevalência dos indicadores de atividade física e comportamento sedentário em adultos das capitais brasileiras entre 2006 e 2021, incluindo o período de pandemia.

**Métodos:**

Trata-se de estudo de série temporal de inquéritos transversais baseado no Sistema de Vigilância Telefônica de Doenças Crônicas. As tendências de atividade física suficiente no lazer, atividade física suficiente no deslocamento, prática insuficiente de atividade física e tempo total de tela foram estimadas por meio da regressão de Prais-Winsten. As tendências temporais e as prevalências anuais foram calculadas por sexo, faixa etária e escolaridade.

**Resultados:**

Na população total, foram encontradas tendências temporais significativas para atividade física suficiente no lazer (β=0,614) e tempo total de tela (β=1,319). Quanto à prevalência, a atividade física suficiente no lazer aumentou de 29% em 2009 para 39% em 2019, seguida de redução de 2,3% entre 2020 e 2021. A prevalência do tempo total de tela aumentou consideravelmente entre 2019 e 2020 (4,7%). Embora a inatividade física tendesse a diminuir ao longo da série, sua prevalência aumentou 3,4% entre 2019 e 2021, assim como a atividade física no deslocamento diminuiu 3,7% no mesmo período.

**Conclusão:**

Enquanto a atividade física suficiente no lazer aumentou ao longo dos anos, é incerto se essa tendência se manterá nos anos seguintes à COVID-19. Não apenas as pessoas alteraram seus hábitos de lazer, mas também há um domínio crescente do tempo de tela em razão da mudança nos padrões sociais e de trabalho. Mais estratégias precisam ser abordadas para o enfrentamento da inatividade física, do comportamento sedentário e para a revisão das metas nacionais pós-pandemia.

## INTRODUCTION

The first case of corona virus disease (COVID-19) in Brazil was confirmed on February 26^th^, 2020, followed by the first community transmission reported in São Paulo, on March 10^th1^. The COVID-19 pandemic magnified existing socioeconomic disparities and health inequities, especially in low- and middle-income countries^2^. Social distancing and isolation measures induced change in the routine of people and families, with alarming implications on the physical and mental health of individuals^3,4^.

The impact of the pandemic on the mitigation and control of non-communicable diseases (NCDs) is a major public health concern^5^. In the context, physical inactivity and sedentary behavior amplify the burden of NCDs, since obesity and chronic conditions are risk factors for the development of severe cases of the diseas^6,7^.

Depicting time trends of physical activity (PA) and sedentary behavior helps to both monitor risk and protective factors for NCDs and to understand the new dynamics of healthy behavior after the COVID-19 pandemic^8^. Previous studies in Brazil indicated an increase in leisure-time physical activity (LTPA) and a reduction in TV-viewing between 2006 and 2012 according to the National Surveillance System for Risk and Protective Factors for Chronic Diseases by Telephone Survey (VIGITEL)^9^, and the same result from 2008 to 2019, based on the Brazilian Health Survey (PNS) and the National Household Sample Survey (PNAD)^10^. However, further studies showed that although time trends of PA increased between 2006 and 2014 at a steady level, they showed gradual reductions after 2016^8^.

After the pandemic, not only has PA reduced at rapid rates, but also sedentary behavior has increased^11^. In Brazil, a cross-sectional study conducted in 2020 showed that ≥4 hours/day of TV-viewing, ≥4 hours/day of computer/tablet use, and physical inactivity increased by 266, 38 and 26%, respectively, in the country^12^. Besides, by drastically raising the number of physically inactive individuals, the COVID-19 pandemic increased the chances of a cardiovascular event, especially among those with preexisting conditions^4^. In fact, while the current Global Plan aims at a 15% reduction in physical inactivity globally, and the Brazilian Action Plan aims at a 30% increase in PA levels, it is still uncertain whether current efforts will compensate for the observed deceleration progress or if the targets will need to be revised^13^.

After COVID-19, much more has been highlighted on the need for continuous surveillance and action planning for the most vulnerable and at high-risk groups^6,14^. To ensure the continuity of care, some strategies have been used, from telemedicine and triage^5^ to incentives for home-based exercises^15^.

In this sense, it is important to monitor the practice of PA in the Brazilian population, aiming to support surveillance, prevention and health promotion actions. In view of this, the objective of this study was to analyze the prevalence and time trends of PA and sedentary behavior indicators in the adult population of the Brazilian state capitals between 2006 and 2021, including the pandemic period.

A closer monitoring of population behavior as regards risk and protective factors for NCDs over the years, with special focus on the prevalence between 2020 and 2021, may elicit a new perspective of action in the context of the novel coronavirus. This is an opportunity to understand if the target of 30% reduction by 2030 in physical inactivity stated in the Brazilian Action Plan to tackle the rise of NCDs^16^ can be achieved or must be revised and, in either case, what can be done to stay on the right course.

## METHOD

### Design and sampling

This is a cross sectional time-series study on PA indicators between the years 2006 and 2021, based on the information from VIGITEL.

VIGITEL is a population-based survey that monitors risk and protective factors for NCDs since 2006 by means of a probabilistic sampling methods that include adults aged 18 years or older living in households with at least one landline telephone in the 26 state capitals of Brazil and the Federal District^17^. Each year, VIGITEL interviews approximately 54,000 individuals^17^. In the years 2020 and 2021, the sample size was approximately of 27,000 individuals^17^. Details on the sampling and data collection process are provided in VIGITEL publications^18^.

### Variables

For the present study, four main indicators were analyzed. First, sufficient LTPA. According to the World Health Organization (WHO), a physically active adult is that who practices a minimum of 150 minutes or more of moderate-intensity PA per week or 75 minutes or more of vigorous-intensity PA per week^19^. Which means that individuals are classified as physically active if they achieve either a combination of 30 minutes of moderate-intensity PA on at least 5 days per week, or 25 minutes of vigorous-intensity PA on at least 3 days/week. The indicator is a composite of the questions: “In the last three months, did you practice any type of physical exercise or sport?”, “What is the main type of physical exercise or sport that you practiced?”, “Do you exercise at least once a week?”, “How many days a week do you usually exercise?” and “On the day you exercise, how long does this activity last?”.

Second, sufficient PA while commuting. Physically active individuals while commuting are those who commute to work or school by bicycle or walking for the equivalent of at least 150 minutes of moderate-intensity PA per week, in other words, those who spend at least 30 minutes per day walking or cycling a round trip to work or school at least five days of the week. Questions about commuting to work and/or school include: “Do you walk or cycle to or from work?”, “How much time do you spend to go back and forth on this route (on foot or by bicycle)?”, “Currently, are you attending a course/school or do you take someone to a course/school?”, and “When you go to or return from this course or school, do you walk or cycle?”.

Third, insufficient practice of PA. Insufficient practice of PA considers the number of individuals whose sum of minutes spent either in physical activities in their free time, commuting to work/school and/or in occupational activity does not reach the equivalent of at least 150 minutes of moderate PA per week. This indicator is estimated from the questions already mentioned about LTPA and PA while commuting and from questions on the individual's occupational activity: “In the last three months, have you worked?”, “In your work, do you carry weight or do other heavy activities?”, “In a normal week, how many days do you do these activities at work?” and “When you perform these activities, how long does it usually last?”. For these three indicators, physical activities lasting less than ten minutes were not considered for the purpose of calculating the weekly sum of minutes spent exercising^17^.

Lastly, we calculated total screen time. This represents the percentage of individuals who have the habit of watching television or using a computer, tablet or cellphone for three or more hours per day. This cutoff represents a marker for sedentary behavior among individuals. The indicator takes into account the answers given to the questions “On average, how many hours a day do you usually watch television?” and “On average, how many hours of your free time (excluding work) does the use of a computer, tablet or cell phone take up per day?”.

The following sociodemographic variables were included: sex (male/female), age category (18–24; 25–34; 35–44; 45–54; 55–64 and 65 years or more), education (0–8; 9–11; 12 years or more) and region (North, Northeast, Central-West; Southeast, and South).

### Data analysis

We obtained the prevalence and time trends as reported by a Prais-Winsten regression for the four indicators and presented results by sex, age category, education, and Brazilian region. Time trends were estimated from 2006 to 2021. However, not all indicators could be reported due to the inconsistency of newly added or revised questions in the questionnaire. LTPA was reported between 2009 and 2021, insufficient practice of PA from 2014 to 2021, and total screen time from 2016 to 2021. The pandemic and post-pandemic period started in 2020.

The slope of the Prais-Winsten regression represented the positive or negative tendency in the overall time period (explanatory variable). The outcome variables were the PA and sedentary behavior indicators, and the explanatory variable was the year of the survey. A negative sign of the slope (β) of the line adjusted by the model indicates that the relationship between the indicator and time is decreasing, while a positive slope value represents the average annual increase. The existence of a significant linear trend was considered when the angular coefficient of the model proved to be different from zero for a p≤0.05. The accuracy of the models was evaluated through its R^2^ value. Besides, we evaluated the annual difference among the years and displayed each increasing or decreasing change in the prevalence. The *survey* command was used in the analyses to consider post-stratification weights of the sampling.

The analyses were performed using the Stata Software version 15.1. VIGITEL data are available for public access and use. Ethical clearance was approved by the National Commission for Ethics in Research for Human Beings of the Ministry of Health (Opinion 2.100.213 – CAAE: 65610017.1.0000.0008).

## RESULTS

Our analyses included 784,479 individuals for the entire study period between 2006 and 2021. In general, we observed significant time trends (p<0.05) for LTPA (2009–2021) and total screen time (2016–2021) in all categories. On the other side, the trends of insufficient PA (2014–2021) and PA while commuting (2006–2021) were non-significant for the entire population and for most categories.

LTPA was reported from 2009 to 2021 (Table 1). In the total population, the trend of the indicator increased steadily (β=0.614; p=0.010) from 2009 to 2019 (29.9 to 39.0%). In following years, the prevalence of LTPA decreased to 36.8% in 2020 and to 36.7% in 2021, which means a reduction in the prevalence of 2.3% between 2019 and 2021. There was a significant time trend increase for both men (β=0.488; p=0.018) and women (β=0.790; p=0.003) over the years analyzed, with greater slope for the latter. In general, though, men demonstrated higher prevalence of LTPA than women, irrespectively of the year. Though there was a steady increase in the levels of LTPA practice from 2009 to 2019 for men (from 39.0% in 2009 to 46.7% in 2019) and women (from 22.1% in 2009 to 32.4% in 2019), the prevalence of LTPA decreased considerably for both sexes in the following years. For men, the decrease was of 3.6% and for women it was of 1.1% between 2019 and 2021.

**Table 1:** Prevalence and time trend of sufficient leisure-time physical activity, according to sociodemographic characteristics. VIGITEL, Brazilian capitals, 2009–2021.

		2009	2010	2011	2012	2013	2014	2015	2016	2017	2018	2019	2020	2021	β*	p-value
	Total	29.9	30.1	31.6	33.5	33.8	35.3	37.6	37.6	37.0	38.1	39.0	36.8	36.7	0.614	0.010
Sex	Male	39.0	39.1	40.4	41.5	41.2	41.6	45.6	46.6	43.4	45.4	46.7	44.2	43.1	0.488	0.018
	Female	22.1	22.4	24.0	26.5	27.4	30.0	30.8	29.9	31.5	31.8	32.4	30.5	31.3	0.790	0.003
Age group	18–24	42.7	43.6	44.4	47.6	49.7	50.0	51.4	52.2	49.1	50.6	49.4	47.1	50.6	0.592	0.049
	25–34	33.9	34.3	35.9	39.1	39.3	41.5	45.2	46.0	44.2	45.5	48.5	41.5	42.6	0.857	0.024
	35–44	25.3	26.0	27.5	31.0	29.6	31.2	36.4	35.7	33.8	36.0	36.8	38.0	34.0	0.951	<0.001
	45–54	24.2	24.3	26.5	25.8	27.3	30.1	30.5	30.4	33.7	32.6	34.6	33.0	34.6	0.951	<0.001
	55–64	24.2	24.4	25.5	25.2	26.6	28.4	29.1	29.7	30.0	32.4	31.5	32.1	31.6	0.733	<0.001
	≥65	22.6	20.7	22.5	23.6	22.3	22.8	23.5	22.3	23.3	24.4	24.4	23.9	21.8	0.137	0.061
Education	0–8	19.5	19.6	21.2	21.6	22.0	22.9	25.4	24.5	23.3	24.6	25.8	23.6	22.6	0.336	0.056
	9–11	34.8	34.6	35.3	37.1	37.2	38.5	40.1	40.4	39.7	40.4	39.5	38.0	37.3	0.236	0.292
	≥12	41.6	41.3	42.5	45.4	45.4	47.8	49.6	47.9	47.0	48.1	50.0	46.2	47.3	0.522	0.029
Region	North	31.6	29.9	32.8	37.2	35.1	37.0	41.3	39.0	40.7	42.4	40.7	35.3	39.3	0.712	0.026
	Northeast	29.4	28.9	31.1	33.4	34.5	35.0	36.1	38.1	37.3	41.2	40.4	41.6	39.8	1.085	<0.001
	Central-West	35.5	36.8	34.8	37.4	39.7	38.2	46.8	43.1	45.0	43.4	43.5	43.4	39.3	0.627	0.040
	Southeast	28.0	28.5	30.0	31.1	30.8	34.0	35.2	35.6	33.4	33.6	36.4	32.0	33.5	0.486	0.024
	South	32.6	33.8	35.4	36.8	38.3	37.7	38.3	37.3	39.4	39.8	40.3	40.9	37.7	0.510	0.003

* The accuracy of the model was evaluated through its R^2^ value.

As regards LTPA according to age categories, coefficients were positive and significant for all groups, except for people with 65 years or more, which also represented the smallest positive slope (β=0.137; p=0.06). Prevalence was higher for younger individuals’ groups, and most groups displayed its peak prevalence in 2019, except for those aged 18 to 24 years (50.6% in 2018 and 49.4% in 2019); 35 to 44 years (36.8% in 2019 and 38.0% in 2020); and 55 to 64 years (32.4% in 2018 and 31.5% in 2019).

Concerning LTPA according to education, the greater the number of years of formal education, the higher the coefficient and the prevalence of LTPA. Nevertheless, it was significant only for the group with more than 12 years of schooling (β=0.522; p=0.02). Higher prevalence was observed in 2019 for all educational groups, with a decrease in the following two years of the series.

Lastly, LTPA was analyzed by Brazilian region. Trends demonstrated a significant increase in the practice of LTPA in all of them, especially in the North (β=0.712; p=0.026) and Northeast (β=1.085; p<0.001). The mean prevalence of LTPA ranged between 32.5% in the Southeast and 40.5% in the Central-West. Amongst all, the peak prevalence was reached in 2019 for the North (40.7%), Central-West (43.5%), and Southeast (36.4%); and in 2020 for the Northeast (41.6%) and the South (40.9%), followed by decreases in the prevalence of LTPA in the population of both these regions.

As regards PA while commuting, trends were analyzed from 2006 to 2021 (Table 2). In the period, no significant values were found, neither for the prevalence in the total population (β=-0.018; p=0.924), nor for sex, age, education, or region, except for an important annual difference between 2019 and 2021. In 2019, the prevalence of PA while commuting was 14.1% and in 2021 it was 10.4%, meaning a reduction of 3.7% within this time-period.

**Table 2:** Prevalence and time trend of sufficient physical activity while commuting. VIGITEL, Brazilian capitals, 2006–2021.

		2006	2007	2008	2009	2010	2011	2012	2013	2014	2015	2016	2017	2018	2019	2020	2021	β*	p-value
	Total	10.9	10.7	11.3	17.0	17.9	14.8	14.2	12.1	12.3	11.9	14.4	13.4	14.4	14.1	13.3	10.4	-0.018	0.924
Sex	Male	13.5	12.7	13.5	17.6	17.9	15.1	13.8	12.2	13.0	12.4	15.4	14.2	15.0	14.5	13.8	10.8	-0.119	0.425
	Female	8.7	9.1	9.4	16.5	17.9	14.6	14.5	11.9	11.6	11.6	13.5	12.8	13.8	13.8	12.9	10.0	0.073	0.746
Age group	18–24	11.4	11.3	12.5	19.8	21.0	18.1	16.5	13.8	14.9	11.9	17.6	14.2	16.0	16.7	16.5	13.1	0.073	0.750
	25–34	12.4	12.3	11.8	19.6	20.8	17.2	16.5	12.6	13.7	13.6	14.8	15.1	15.5	14.4	15.2	10.5	-0.095	0.670
	35–44	12.9	13.1	13.9	19.5	21.2	17.1	15.6	15.0	14.3	14.9	17.1	15.9	17.9	16.6	15.5	11.7	-0.034	0.873
	45–54	12.3	11.7	12.5	17.8	19.0	14.6	15.0	13.5	12.7	13.2	15.2	14.9	14.8	17.2	14.8	12.4	0.040	0.804
	55–64	7.1	7.5	9.5	12.0	11.6	10.8	11.3	9.4	9.6	9.2	12.7	11.2	13.0	11.4	9.7	8.9	0.125	0.350
	≥65	3.3	2.3	2.6	4.5	3.9	4.3	4.2	3.0	3.6	4.0	5.0	4.7	5.1	4.8	3.6	3.4	0.068	0.246
Education	0–8	13.4	12.4	12.7	18.5	18.6	15.3	14.5	12.0	12.7	12.3	14.5	14.6	14.9	14.3	12.7	9.2	-0.198	0.302
	9–11	10.3	10.8	11.8	17.7	19.1	15.5	15.2	13.0	13.4	13.0	15.6	14.5	16.0	15.7	14.6	13.1	0.143	0.468
	≥12	6.4	6.8	7.9	13.1	15.0	13.0	12.1	10.8	10.0	10.0	12.9	11.0	11.9	12.2	12.4	8.0	0.128	0.549
Region	North	13.7	13.8	13.6	19.1	18.8	16.2	13.4	11.8	12.1	11.2	13.2	12.4	12.4	12.8	12.7	11.1	-0.245	0.159
	Northeast	11.6	10.9	11.2	16.6	16.4	13.6	13.5	11.2	11.4	10.0	12.9	11.8	12.9	12.4	13.0	9.9	-0.099	0.488
	Central-West	9.8	10.1	9.6	13.6	13.6	11.6	12.3	9.6	8.9	7.0	10.3	11.7	10.5	10.4	8.5	7.7	-0.153	0.272
	Southeast	10.0	10.4	11.5	17.7	20.0	15.7	15.4	13.4	13.7	14.3	16.7	15.4	16.8	16.4	14.7	11.5	0.121	0.611
	South	10.9	10.1	10.1	16.4	15.5	16.4	13.0	11.0	12.1	12.5	13.8	11.5	13.8	14.3	14.4	9.0	-0.007	0.966

* The accuracy of the model was evaluated through its R^2^ value.

The time trend of insufficient physical active adults could be reported only from 2014 to 2021 (Table 3). In this period, one significant value was found for the Northeast Region, in which the indicator showed a considerable decrease (β=-0.804; p<0.007) throughout the years analyzed. In the total population, although no significant values were found, there was an important reduction in the prevalence of physical inactivity between 2014 and 2019, followed by an increase of 2.4% between 2019 and 2020 and of 3.4% between 2019 and 2021.

**Table 3:** Prevalence and time trend of insufficient practice of physical activity. VIGITEL, Brazilian capitals, 2014–2021.

		2014	2015	2016	2017	2018	2019	2020	2021	β*	p-value
	Total	48.7	47.5	45.1	46.0	44.1	44.8	47.2	48.2	-0.086	0.818
Sex	Male	40.1	37.2	34.1	37.6	35.1	36.1	37.3	39.3	-0.019	0.957
	Female	56.0	56.3	54.5	53.1	51.7	52.2	55.6	55.7	-0.093	0.819
Age group	18–24	37.0	37.5	34.3	37.5	35.7	36.5	38.4	35.6	0.060	0.656
	25–34	41.3	38.9	36.7	36.7	35.6	36.8	40.8	42.6	0.189	0.760
	35–44	47.2	44.3	42.3	44.6	40.8	42.2	44.3	45.0	-0.233	0.512
	45–54	51.2	50.0	46.9	46.2	45.2	44.2	44.3	46.3	-0.776	0.057
	55–64	57.3	58.0	53.9	54.1	51.2	52.3	55.7	56.6	-0.210	0.683
	≥65	72.5	71.7	71.2	70.6	69.2	69.1	70.4	73.0	0.004	0.990
Education	0–8	56.9	56.0	53.7	54.9	53.4	53.7	57.5	58.4	0.206	0.618
	9–11	44.9	44.5	41.6	42.9	39.8	43.4	44.1	45.2	0.035	0.921
	≥12	42.9	41.0	40.2	40.8	40.3	38.6	42.3	43.5	0.065	0.837
Region	North	48.4	46.5	44.9	45.6	44.1	45.2	48.6	46.8	-0.059	0.854
	Northeast	50.1	51.0	46.0	48.2	44.1	45.8	44.3	47.2	-0.804	0.007
	Central-West	46.1	41.8	41.2	40.6	40.9	42.5	44.2	45.2	-0.006	0.990
	Southeast	46.1	41.8	41.2	40.6	40.9	42.5	44.2	45.2	-0.006	0.990
	South	46.7	46.5	46.6	44.5	42.2	42.8	43.0	48.0	-0.089	0.852

* The accuracy of the model was evaluated through its R^2^ value.

The habit of staying in front of a screen, measured as total screen time, was analyzed between 2016 and 2021 (Table 4). The time trends were positive for all except one category, that of individuals aged 18 to 24 years (β=0.445; p=0.23). In the total population, the increase was of 1.319 over the years (p=0.001), and higher for women (β=1.499; p=0.004) than for men (β=1.099; p<0.001); for individuals aged 45 to 54 years (β=2.224; p<0.001) and 55 to 64 years (β=2.312; p=0.002) than other age groups; people with 0 to 8 years of education (β=1.577; p<0.001) than those with more years of schooling; and in the Central-West (β=1.718; p=0.001) and Southeast (β=1.520; p<0.001) among all regions. The largest increase in total screen time was observed between 2019 and 2020 for all categories, meaning a greater annual variation in those years (Table 5).

**Table 4:** Prevalence and time trend of total screen time. VIGITEL, Brazilian capitals, 2016–2021.

		2016	2017	2018	2019	2020	2021	β*	p-value
	Total	61.7	61.0	63.3	62.7	67.4	66.0	1.319	0.001
Sex	Male	62.9	62.1	65.0	63.9	67.3	66.7	1.099	<0.001
	Female	60.6	60.1	61.9	61.7	67.5	65.4	1.499	0.004
Age group	18–24	82.1	79.9	81.3	79.2	83.3	83.2	0.445	0.238
	25–34	73.9	71.7	74.3	73.3	78.1	73.9	0.903	0.010
	35–44	59.2	60.7	62.8	62.4	66.1	64.6	1.353	<0.001
	45–54	51.1	50.8	55.5	53.9	60.3	60.2	2.224	<0.001
	55–64	48.2	48.8	50.5	52.1	58.6	57.0	2.312	0.002
	≥65	42.3	42.5	43.8	45.7	49.3	51.0	1.848	0.003
Education	0–8	45.1	44.3	48.3	46.3	52.7	49.2	1.577	<0.001
	9–11	69.3	67.4	69.6	68.5	72.7	71.3	0.880	0.011
	≥12	70.1	69.6	70.1	70.2	73.3	73.0	0.754	0.024
Region	North	62.8	62.3	64.4	63.2	67.4	66.2	1.025	0.001
	Northeast	60.7	61.1	62.1	61.6	65.2	64.5	0.945	0.003
	Central-West	58.7	58.3	61.1	60.4	66.2	64.8	1.718	0.001
	Southeast	62.9	61.2	64.6	64.0	69.1	66.8	1.520	<0.001
	South	61.0	61.5	61.7	61.2	66.5	67.5	1.339	0.031

* The accuracy of the model was evaluated through its R^2^ value.

**Table 5:** Annual difference in the total prevalence of physical activity indicators. VIGITEL, Brazilian capitals, 2006–2021.

Year	LTPA		PA while commuting		Insufficient PA practice		Total screen time	
	Prevalence (%)	Annual difference	Prevalence (%)	Annual difference	Prevalence (%)	Annual difference	Prevalence (%)	Annual difference
2006	*	*	10.9	*	*	*	*	*
2007	*	*	10.7	-0.2	*	*	*	*
2008	*	*	11.3	0.6	*	*	*	*
2009	29.9	*	17.0	5.7	*	*	*	*
2010	30.1	0.2	17.9	0.9	*	*	*	*
2011	31.6	1.5	14.8	-3.1	*	*	*	*
2012	33.5	1.9	14.2	-0.6	*	*	*	*
2013	33.8	0.3	12.1	-2.1	*	*	*	*
2014	35.3	1.5	12.3	0.2	48.7	*	*	*
2015	37.6	2.3	11.9	-0.4	47.5	-1.2	*	*
2016	37.6	0.0	14.4	2.5	45.1	-2.4	61.7	*
2017	37.0	-0.6	13.4	-1.0	46.0	0.9	61.0	-0.7
2018	38.1	1.1	14.4	1.0	44.1	-1.9	63.3	2.3
2019	39.0	0.9	14.1	-0.3	44.8	0.7	62.7	-0.6
2020	36.8	-2.2	13.3	-0.8	47.2	2.4	67.4	4.7
2021	36.7	-0.1	10.4	-2.9	48.2	1.0	66.0	-1.4

LTPA: leisure-time physical activity; PA: physical activity;

* data were not available during this period.

## DISCUSSION

The study analyzed the annual prevalence and time trends of PA indicators among Brazilian adults, from 2006 to 2021. Generally, before the pandemic period people became more physically active, as we can see by both an increase in LTPA practice and a reduction in insufficient PA. However, the overall increase over the years was disrupted by a drop in LTPA and an increase in insufficient PA after 2019. Additionally, it was observed a reduction in sufficient PA while commuting and an increase in total screen time, also accentuated after 2019. In general, LPTA trends were lower for people aged 65 years or more, for women, and for people with lower education.

Two observations must be highlighted. Firstly, the prevalence of PA and sedentary behavior demonstrated a shift between 2019 and 2021. The decreasing prevalence of LTPA and increasing prevalence of insufficiently active individuals between these years could be most probably associated with the COVID-19 pandemics and the change in behavior dynamics^20^. It is noteworthy that, in Brazil, healthy behavior decreased after the COVID-19 pandemic not only for total population^21^, but especially for those who reported some type of NCD, such as diabetes, hypertension, heart disease, cancer or respiratory diseases^22^. In fact, during the pandemic, despite incentives for home-based exercises to maintain PA levels^15^, people were most of the time reclused due to social isolation and mitigation measures to control the spread of the virus, impacting on the control of such chronic conditions^20^.

Likewise, PA while commuting had its largest decrease between 2019 and 2021, by 3.7%, and total screen time increased considerably by 3.3% in the same period. Alterations in total screen time could be related to the reduced options of recreational activities during lockdown as well as to the increasing distress caused by the general context^23^, which also negatively influenced sleeping in all age categories^24^ but specially for children and adolescents^23,25^. Over the years, total screen time increased more among women, older adults and people with fewer years of formal education, supposedly due to the spread of digitalization and increased access to technology. Other studies found a reduction in TV-viewing among people with higher education and younger age, but the measure did not include other screen devices such as computer, cellphone and tablets, which have been replacing TV-viewing^10^. During the COVID-19 pandemic, higher increases in the prevalence of solely TV-viewing was observed among younger adults and those with higher schooling, but the prevalence remained higher for older adults and individuals with fewer years of formal education^12^.

Secondly, there might be an interesting point on motivation as a determinant for healthy behavior adherence. Whereas men, people of younger age and those with more years of education tend to exercise more and show less sedentary behavior, LTPA prevalence reduced more among these same groups between 2019 and 2021, which was also observed previously^12^. However, this is surprising because it would be expected for them to maintain the same pattern. Such observations are important and need further detailing so as to better understand the determinants of health and sedentary behavior during the pandemic^26^ as well as inequities in PA practice^27^.

Results suggest that the COVID-19 pandemic has altered PA patterns in the population and in the cities^28^. Added up by the misaligned sum of political forces to deal with the situation^29^, one of the effects of the pandemic was not only that people reduced their time outside and changed habits by staying longer periods in front of screens, but also that the demographic and epidemiological transitions together with the increasing dominance of technology in working and social environments might have intensified the observed pattern^11^.

On one side, while the North and the Northeast regions are the most vulnerable in the country^30^, they presented the highest trends of LTPA practice and the lowest total screen time. Although such tendencies contradict that lower socioeconomic status predicts lower PA outcomes, access to primary health care and social assistance programs tend to be higher in these regions^30^, pointing to the importance of government-level support to promote populational protective behaviors.

This is a cross-sectional study which reveals relevant tendencies on NCD risk and protective factors along the years. Though correlations with contextual factors can be stated, we understand they cannot directly prove a cause-effect relationship. Our findings are conservative and based on evidences from previous studies showing that the COVID-19 pandemic led to drawbacks on healthy behavior against the rise of NCDs^11,12,20–23^. Additional misaligned government response may have influenced the increasing disparities in PA and sedentary behavior during the pandemic^29,30^.

To our knowledge, this study sheds light on the need to further investigate the impact of the COVID-19 pandemic on the determinants of PA as well as on the global and national targets in different scenarios. With regards to the limitations of the findings, the insignificant statistics observed for sufficient PA while commuting and for most categories of insufficient PA practice could be related to the data errors, which still do not account for the complete time-series. Also, the lack of consistency remaining in the questionnaires reveal a demand for standardization in surveillance methods across the years. VIGITEL collects self-declared data by landline and the use of post-stratification weights aims to reduce representation bias. Nevertheless, with the reduction of landline coverage, the non-representation of the population may increase. Besides, VIGITEL is not representative of the entire country, but only of the adult population of Brazilian state capitals.

In order for Brazil to continue on the track of the National target of a 30% increase in the prevalence of LTPA until 2030 and the goal of 15% decrease in physical inactivity as stated in the Global Agenda, we highlighted that more government-level strategies have to be addressed in order to reduce the downward tendency observed in the past years. Revalidation of the global and national targets is also an action to be ruled. Population levels of PA practice and sedentary behavior are still a challenge, confronted by new life perspectives after the COVID-19 pandemic.
